# NK cell-related genes-driven novel molecular subtyping and prognostic signatures for Wilms tumor: uncovering the therapeutic potential of TGX-221 and biomarker role of HS2ST1

**DOI:** 10.3389/fonc.2025.1593011

**Published:** 2025-08-28

**Authors:** Peng Hong, Zaihong Hu, Jie Lin, Kongkong Cui, Zhiqiang Gao, Xiaomao Tian, Tao Lin, Qinlin Shi, Guanghui Wei

**Affiliations:** ^1^ Department of Urology, Children’s Hospital of Chongqing Medical University, Chongqing, China; ^2^ National Clinical Research Center for Child Health and Disorders, Ministry of Education Key Laboratory of Child Development and Disorders, Chongqing, China; ^3^ Chongqing Key Laboratory of Structural Birth Defect and Reconstruction, Chongqing, China

**Keywords:** Wilms tumor, natural killer cells, molecular subtyping, prognostic signatures, TGX-221, HS2ST1

## Abstract

**Background:**

Wilms tumor (WT) lacks precise molecular subtyping tools, which limits the development of personalized therapies. To address this issue, we investigated whether NK cell-related genes (NKGs) could refine the molecular subtyping of WT, aiming to identify novel therapeutic strategies.

**Methods:**

Consensus clustering was employed for the molecular subtyping of WT. The immune microenvironment of different WT subtypes was assessed using immune profiling algorithms. Potential therapeutic compounds targeting the identified subtypes were screened using the CMap database, and their mechanisms of action were elucidated through molecular docking and molecular dynamics simulations. Subsequently, *in vitro* cell experiments, including CCK8, flow cytometry, and Transwell assays, were performed to assess the biological behavior of tumor cells. A prognostic signatures was constructed using machine learning algorithms, with its performance evaluated by ROC curves, calibration curves, and the concordance index. Additionally, cellular localization and expression of marker genes were investigated through single-cell analysis and validated using RT-qPCR.

**Results:**

We developed novel molecular subtyping tools that classified WT into prognostically distinct subtypes: “immune-rich” and “immune-desert”. Screening the CMap database identified the small-molecule drug TGX-221 as a candidate modulator. TGX-221 significantly inhibited the malignant progression of WT through a dual-action mechanism: blocking the key oncogenic Wnt/β-catenin signaling pathway and sensitizing tumor cells to NK cell-mediated cytotoxicity. Furthermore, a prognostic signatures based on HS2ST1, EPI3M, and PPP3CA effectively predicted patient outcomes. Notably, HS2ST1 emerged as a novel biomarker, potentially promoting cancer stem cell-like properties via heparan sulfate-mediated enhancement of Wnt/β-catenin signaling, highlighting its dual value as both a prognostic indicator and a therapeutic target.

**Conclusion:**

Molecular subtyping and prognostic signatures based on NKGs enable the precise identification of high-risk WT patients. Moreover, TGX-221 represents a promising novel therapeutic candidate, while HS2ST1 serves as a potential prognostic biomarker. These findings collectively provide tools for risk stratification and targeted therapy, advancing precision oncology for WT.

## Introduction

Nephroblastoma, also designated as Wilms tumor (WT), the most prevalent pediatric renal malignancy, remains incompletely elucidated in its pathogenic mechanisms (1–3). Prevailing theories suggest its origin lies in the developmental arrest of embryonic nephrogenic progenitor cells, where synergistic genetic alterations (such as WT1/2 deletions) and epigenetic dysregulation conspire to reactivate primitive signaling cascades such as Wnt/β-catenin (1, 4–7). This molecular reprogramming ultimately subverts microenvironmental homeostasis, driving uncontrolled proliferation (1, 4, 5). Epidemiologic patterns demonstrate striking geographic heterogeneity, implicating gene-environment interactions in disease initiation (8–10). Histopathologically, three distinct subtypes are recognized: the classical triphasic pattern, anaplastic variant with hallmark nuclear pleomorphism, and the recently characterized desert subtype featuring TP53 mutations with concomitant cGAS-STING pathway inactivation (11). Crucially, these high-risk subtypes (anaplastic and desert), though constituting only 15%-20% of total cases, account for 90% of disease-related mortality, highlighting the imperative for molecular subtyping in therapeutic stratification (12).

While contemporary multimodal therapies have elevated the 5-year survival rate beyond 90% in WT patients, critical challenges persist: approximately 20% of patients develop chemoresistance-driven treatment failure (2, 13, 14). In comparison, 40% of survivors endure chemotherapy-associated late effects including secondary malignancies and organ dysfunction (13). The therapeutic dilemma between radical resection and nephron-sparing preservation remains unresolved (15). Recent molecular subtyping studies have uncovered compelling associations between tumor microenvironment remodeling - particularly the immune/stromal depletion signature characteristic of desert subtypes - and therapeutic resistance (11, 16–18). These findings underscore the imperative of developing biomarker-guided risk stratification systems and targeted interventions. Consequently, elucidating tumor heterogeneity and engineering novel therapeutic modalities to overcome drug resistance have emerged as pivotal research frontiers for improving outcomes in high-risk cohorts.

The tumor immune microenvironment (TME) constitutes a dynamic ecosystem comprising neoplastic cells, immune populations (T cells, Natural Killer cells, macrophages), stromal components, extracellular matrix, and signaling molecules (cytokines, metabolites) (19). This interplay orchestrates tumor progression through immune evasion, neoangiogenesis, and metabolic reprogramming. Notably, pediatric solid tumors differ fundamentally from their adult counterparts by exhibiting attenuated tumor antigen presentation and innate immunity-dominant TME profiles, contrasting with the mutation-driven T cell-infiltrated landscapes characteristic of adult malignancies (20, 21). NK cells, as pivotal effectors of innate immunity, mediate tumor surveillance through granzyme/perforin-dependent cytotoxicity, antibody-dependent cellular cytotoxicity, and interferon-γ/TNF-α secretion (22, 23). Emerging evidence indicates that post-chemotherapy NK cell functional reconstitution demonstrates significant positive correlations with therapeutic responses in neuroblastoma (24, 25). Notably, umbilical cord blood-derived NK cells demonstrate metastasis suppression efficacy comparable to conventional chemotherapy in WT preclinical models (25–27). These collective insights position NK cell adoptive immunotherapy as a promising therapeutic frontier for high-risk WT management.

The dynamic equilibrium between activating receptors (e.g., NKG2D) and inhibitory receptors (e.g., Siglec-9) plays a pivotal role in regulating NK cell functional activity (28). Our preliminary studies demonstrated that combined IL-2/IL-15 intervention activates the MAPK signaling pathway, effectively upregulating NKG2D expression and enhancing NK cell-mediated cytotoxicity against WT (29). Furthermore, the immunoregulatory function of the Siglec-9/Siglec-9L axis in hepatocellular carcinoma immune evasion has been increasingly validated, alongside the clinical prognostic value of NK cell-related gene signatures in glioblastoma (30). However, critical knowledge gaps persist regarding the mechanistic role of NK cell-associated molecular markers in the molecular subtyping of WT (31). Systematic elucidation of this mechanism will establish a theoretical foundation for developing an NK cell functionality-based molecular subtyping system. This advancement is expected to guide the design of precision immunotherapeutic strategies targeting high-risk WT subtypes, addressing current limitations in conventional therapeutic paradigms.

**Figure 1:**
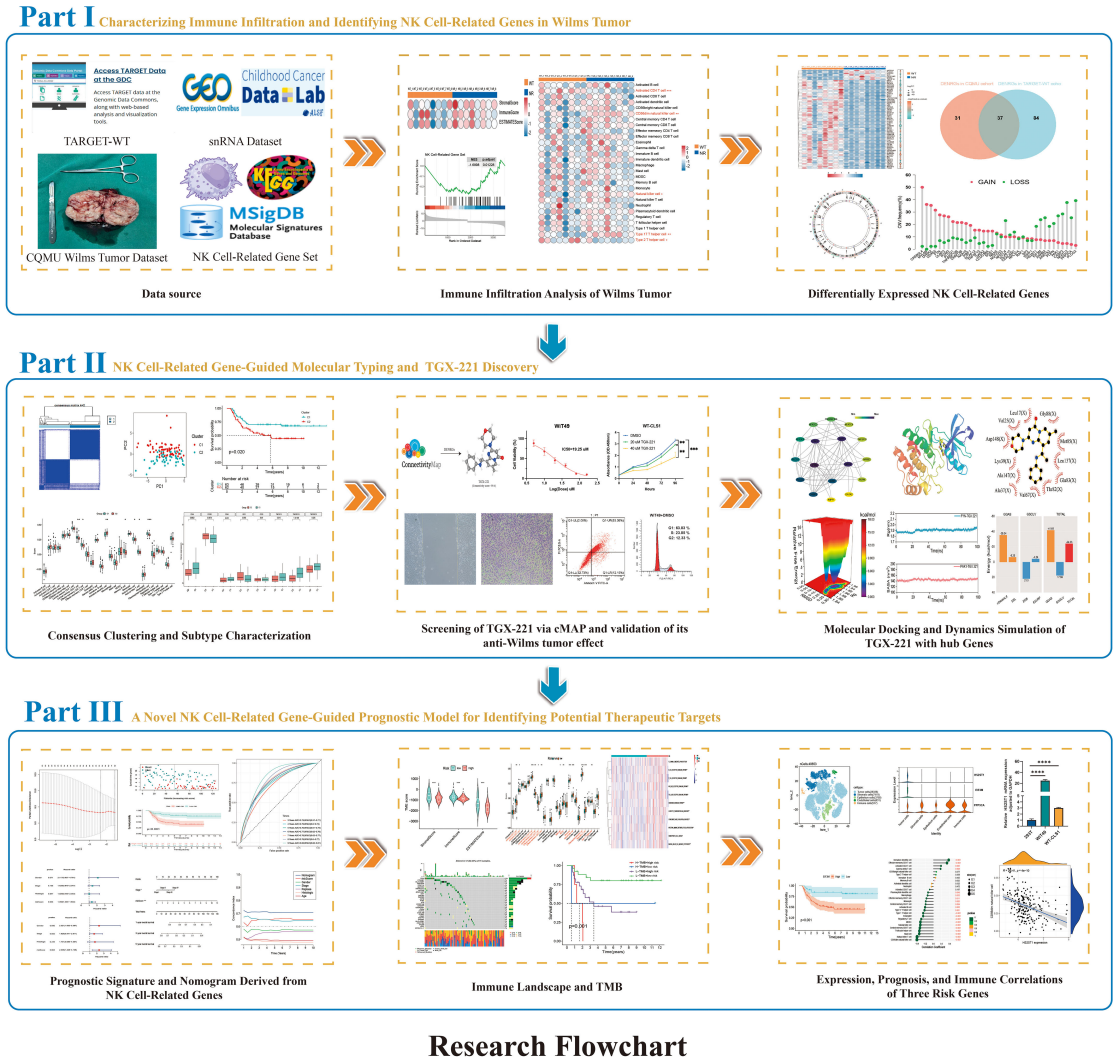
Graphical representation of the study.

In this study, we systematically characterized the molecular heterogeneity of WT through integrated multi-omics sequencing technologies encompassing transcriptome profiling and single-cell sequencing. For the first time, a novel molecular subtyping framework based on consensus clustering was established from NK cell-associated molecular dimensions in WT. Validation across multi-center datasets revealed significant inter-subtype survival disparities and immune microenvironment heterogeneity. Through computational biology approaches, we identified candidate drugs with subtype-reversing potential, subsequently validating their mechanisms via molecular docking and dynamics simulations. The developed prognostic prediction model provides a quantitative tool for clinical decision-making. This investigation expands the theoretical framework of WT molecular subtyping from an NK cell perspective, while the discovered biomarkers and potential therapeutic strategies lay a crucial foundation for precision medicine. **Figure 1** illustrates the systematic research workflow in flowchart format, encompassing data acquisition, bioinformatics analysis, and experimental validation.

## Materials and methods

### Multi-omics data acquisition

The CQMU cohort (GEO accession GSE197047) comprises RNA sequencing data from 8 paired WT tissues and matched normal kidney tissues, enabling paired analytical design (32). The TARGET-WT dataset provides multidimensional data from 125 WT cases and 6 normal kidney controls, including clinical parameters, transcriptomic profiles, somatic copy number variations (CNV), and single-nucleotide variations (33). GSE31403 contributes RNA expression profiles of 224 WT samples generated using Affymetrix microarray chips. Single-cell sequencing data encompassing 6 WT samples were obtained from the Single-cell Pediatric Cancer Atlas Portal (https://scpca.alexslemonade.org/projects/SCPCP000006). Additionally, leveraging the ImmPort, TISDIB, and KEGG databases alongside published literature, we constructed a gene set containing 329 NK cell-related genes (NRGs). Specifically, the gene set was built by: (1) extracting genes annotated with NK cell-specific functions or markers from the ImmPort database; (2) retrieving genes from functional modules in the TISIDB database, including molecular signatures related to “Natural killer cell” (e.g., NK cell activation, NK cell-mediated immune response to tumor cells), ensuring that all relevant signatures were systematically incorporated during the retrieval process; (3) including all genes in the KEGG database for the “Natural killer cell mediated cytotoxicity” pathway (hsa04650); and (4) supplementing this with genes identified through systematic literature mining in PubMed using the key phrase “NK cell-related gene” in titles and abstracts, followed by a manual review. This rigorous process yielded a total of 329 high-confidence NRGs. We further validated the functional relevance of these genes through gene enrichment analysis. The complete gene set is provided in **Supplementary File 1** (31, 34, 35).

### Differential gene expression analysis

Differentially expressed genes (DEGs) were identified using the R package “limma”. Linear models were constructed for individual genes through the lmFit function, followed by empirical Bayes adjustment implemented via the eBayes function (36). Candidate DEGs were selected under stringent thresholds (false discovery rate-adjusted *P* < 0.05 and |fold change| ≥ 2). To validate analytical rigor, we applied Benjamini-Hochberg false positive rate correction, with subsequent visualization of gene expression patterns through volcano plot and heatmap.

### Consensus clustering

As an unsupervised ensemble clustering method, consensus clustering generates multiple clustering results through repeated random subsampling and clustering operations (37). These results are then aggregated to construct a consensus matrix, which evaluates the stability and validity of clustering patterns. Specifically, we utilized the “ConsensusClusterPlus” package with the Pearson correlation distance metric and hierarchical clustering algorithm. By calculating consensus matrices and silhouette coefficients under different cluster numbers (k values), the optimal number of clusters was determined, ultimately stratifying the samples into distinct tumor subtypes.

### Survival analysis

Kaplan-Meier survival curves with log-rank tests were plotted to compare survival differences between patient groups stratified by gene expression levels (38). Univariate Cox proportional hazards regression models were employed to assess the prognostic effects of gene expression levels and clinical characteristics (e.g., age, gender, tumor stage), with results presented as hazard ratios and corresponding 95% confidence intervals, where statistical significance was defined as *P* < 0.05. Subsequently, multivariate Cox regression analysis was performed by incorporating significant variables identified in univariate analysis, aiming to evaluate their independent prognostic value.

### Immune analysis

The ESTIMATE algorithm was utilized to calculate StromalScores, ImmuneScores, and ESTIMATEScores for each sample, evaluating the infiltration levels of stromal and immune cells in tumor tissues (39). Single-sample gene set enrichment analysis (ssGSEA) was performed to quantify the infiltration levels of different immune cell subtypes based on gene sets defining 28 immune cell subtypes established by Zlatko Trajanoski et al., with subsequent comparison of their relative abundance (40). Additionally, expression levels of common immune checkpoint-related genes were extracted to analyze inter-sample variations and assess immune checkpoint activity. Finally, the activity of immune-related pathways in different samples was evaluated by calculating the expression levels of key genes within each immunologically relevant pathway.

### Computational drug screening

The CMap database serves as a computational platform for drug candidate discovery by matching disease-specific differential gene expression profiles with transcriptional signatures of cells exposed to known small molecules, thereby identifying compounds capable of reversing disease-associated gene expression patterns (41). The Kolmogorov-Smirnov enrichment algorithm was employed to quantify inverse correlations between input gene sets and transcriptomic signatures of 1,309 pharmacologically perturbed cellular states, generating connectivity scores ranging from -100 (maximum reversal potential) to +100 (maximum synergy). Compounds demonstrating statistically significant negative connectivity scores (absolute value >90, *P*<0.05) were prioritized as putative phenotype-reversing agents (41). Based on this computational framework, the top-ranked compounds with the most negative connectivity scores were selected as potential therapeutic candidates for further investigation.

### Molecular docking and molecular dynamics simulations

Using AutoDock Tools 1.5.6 for protein hydrogen addition and small-molecule hydrogen addition and torsion bond definition. The Grid module was used to generate the grid box for docking. Docking was performed in AutoDock Vina 1.2.0 with flexible side chains of the small molecule (exhaustiveness = 25). For molecular dynamics simulations (Gromacs 2024.4), the Amber14sb and GAFF2 force fields were applied to the protein and ligand, respectively, with solvation in a TIP4P water model (1.2 nm periodic boundary box). Long-range electrostatics were handled via PME, and the system was neutralized with Na^+^/Cl^-^ ions using the Monte Carlo method. Energy minimization involved 50,000 steepest descent steps (max force <1000 kJ/mol), followed by 50,000-step NVT (310 K) and NPT (1 atm, 310 K) equilibrations (2 fs timestep). A 100 ns production simulation (2 fs timestep) was conducted, saving coordinates every 10 ps. Trajectory analysis included RMSD, RMSF, Rg, hydrogen bond counts, free energy profiles, structural comparisons at 0/25/50/75/100 ns, and MM/GBSA binding free energy calculations.

### Prognostic signatures and nomogram construction

Prognostic genes were initially screened via univariate Cox regression analysis. Subsequent feature dimension reduction was implemented using LASSO regression (10-fold cross-validation, regularization parameter λ selection under the one standard error rule). The risk score formula was derived as: Risk Score = Σ(LASSO coefficient × gene expression level). By integrating the risk score with clinically independent prognostic factors, a nomogram was developed to visually demonstrate the prognostic contributions of variables and quantitatively predict survival probability. Model discrimination was assessed through the concordance index (C-index), with prediction accuracy evaluated via calibration curves (1000 resamplings). Clinical net benefit was quantified using decision curve analysis (DCA), while predictive performance was determined by time-dependent receiver operating characteristic (ROC) curves, where the area under the curve (AUC) measured classification capability. All analyses were implemented using R packages glmnet, rms, ggDCA, and timeROC.

### Single-cell data analysis

The single-cell transcriptomic data were analyzed through a standardized pipeline encompassing data quality control, batch effect correction, dimensional reduction, clustering, and cell-type annotation. Initially, rigorous quality control was performed on the raw sequencing data to filter high-quality cells for downstream analyses. Subsequently, the Harmony algorithm was applied to eliminate batch effects across experimental batches, ensuring data comparability. Dimensionality reduction was achieved via principal component analysis, followed by visualization using either Uniform Manifold Approximation and Projection or t-distributed stochastic neighbor embedding to discern cellular distribution patterns. Cell clustering was conducted using graph-based algorithms on the reduced-dimensional space to identify distinct subpopulations. Final cell-type annotation was performed by cross-referencing canonical marker genes with established biological databases to determine cellular identities.

### *In vitro* cell experiments

The WIT49 and WT-CLS1 WT cell lines (ATCC-derived) were maintained at the Chongqing Key Laboratory of Structural Birth Defects and Organ Reconstruction. TGX-221 (HY-10114, MedChemExpress) was dissolved in DMSO and assessed for cytotoxicity via CCK-8 assays (HY-K0301), where cells seeded at 5×10³/well in 96-well plates were treated with gradient concentrations, incubated for specified durations, and measured at 450 nm to determine IC50 values. Cell migration and invasion capacities were evaluated through wound healing assays (scratch closure monitored at 0/48 hours) and Matrigel-coated Transwell chambers (Corning^®^), respectively, with invaded cells quantified after crystal violet staining. Apoptosis and cell cycle distribution were analyzed by flow cytometry using Annexin V-FITC/PI double staining and PI single staining, while qRT-PCR profiling with Tsingke Biotechnology-synthesized primers evaluated gene expression normalized to GAPDH via the 2^-ΔΔCt method.

### Statistical analysis

All statistical analyses in this study were performed using R (version 4.3.1) and GraphPad Prism (version 9.5) software.

## Results

### Characterization of the immune microenvironment and identification of NKGs in WT

**Figure 2:**
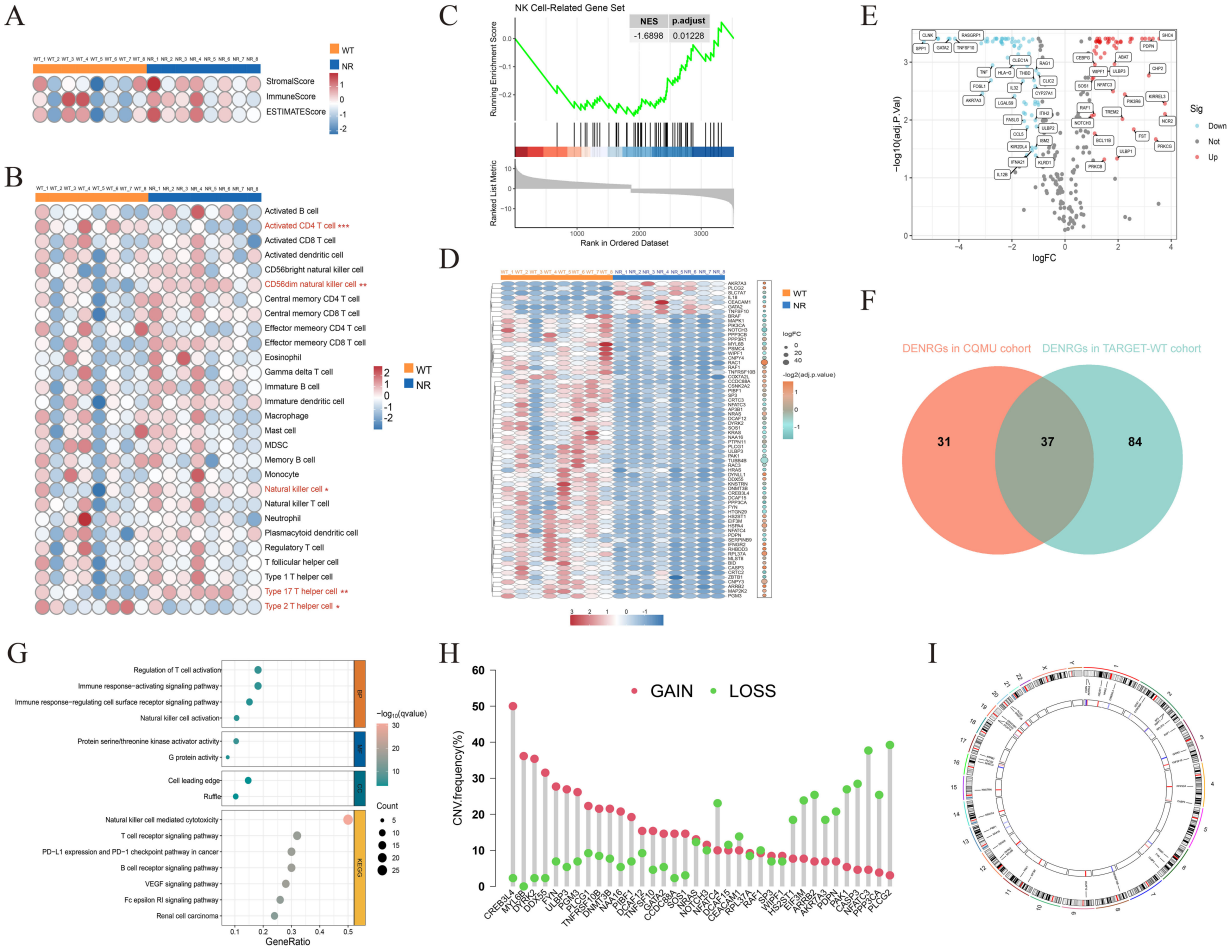
Analysis of the immune microenvironment in Wilms tumor and identification of NK cell-related genes. ESTIMATEScore, ImmuneScore, StromalScore, **(A)** and infiltration levels of 28 immune cell subtypes **(B)** were evaluated in 8 pairs of Wilms tumors and adjacent normal kidney tissues. **(C)** GSEA analysis showing the downregulation of NK cell-related gene sets in Wilms tumor (NES = -1.6898, *p*.adjust = 0.01228). **(D)** The heatmap illustrates the expression of 68 differentially expressed NK cell-related genes in the CQMU cohort. **(E)** The volcano plot illustrates that 121 out of 329 NK cell-related genes are differentially expressed in the TARGET-WT cohort. **(F)** The Venn diagram shows the overlap between the CQMU cohort (68 genes) and the TARGET-WT cohort (121 genes), with 37 differentially expressed NK cell-related genes identified in both cohorts. Functional enrichment analysis **(G)**, copy number variation analysis **(H)**, and chromosomal location distribution **(I)** of 37 differentially expressed NK cell-related genes.

We analyzed transcriptomic data from eight paired samples of WT and adjacent normal renal tissues in the CQMU cohort, using ESTIMATE and ssGSEA algorithms. WT exhibited significantly reduced ImmuneScore compared to adjacent normal renal tissues (**Figure 2A**), with a prominent decrease in CD56dim NK cell infiltration (**Figure 2B**). By leveraging 329 previously reported NRGs, we constructed an NK-related gene set. GSEA analysis revealed that the activity of NK cell-related pathways significantly differs between WT and adjacent normal renal tissues (**Figure 2C**). Subsequent analysis identified 68 differentially expressed NRGs in the CQMU cohort (**Figure 2D**), which were further validated by differential expression analysis in the TARGET-WT cohort (**Figure 2E**). The intersection of these datasets yielded 37 differentially expressed NRGs (**Figure 2F**), with functional enrichment showing a strong association with NK cell activation and immune responses (**Figure 2G**). CNV analysis demonstrated frequent copy number gains (e.g., CREB3L4, MYL6B) and losses (e.g., PPP3CA, PLCG2) in these genes (**Figure 2H**), and their chromosomal location distributions were mapped (**Figure 2I**). These findings reveal the abnormal characteristics of NK cells in the immune microenvironment of WT and suggest that these differentially expressed NRGs may be associated with WT progression, although further functional studies are needed to confirm their roles as potential drivers.

### Unsupervised clustering based on 37 differentially expressed NRGs characterizes WT subtypes

**Figure 3:**
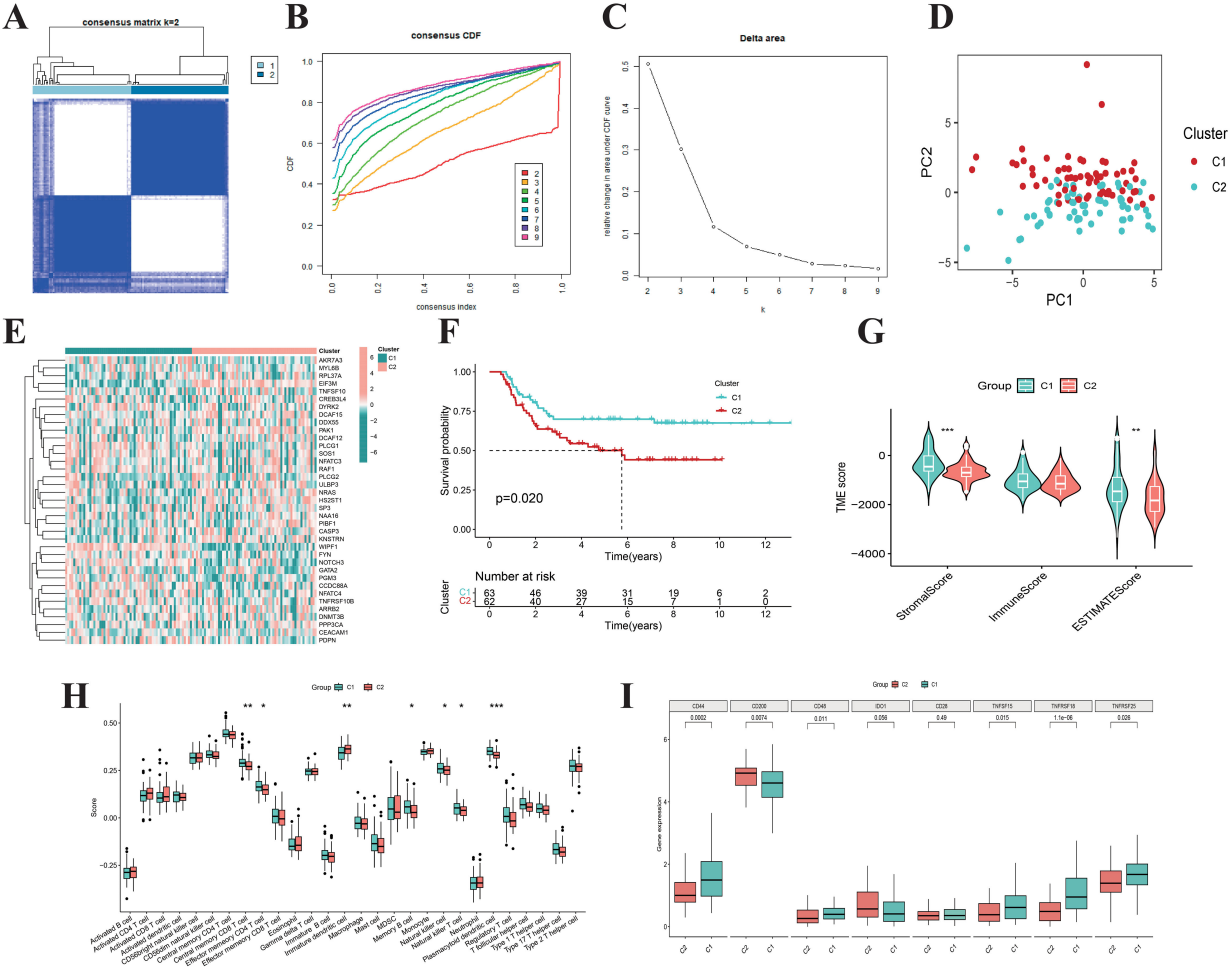
Consensus clustering. **(A)** When k = 2, the Wilms tumor samples are divided into two distinct clusters. The cumulative distribution function **(B)** and Delta area plot **(C)** validate the stability and rationality of clustering at K = 2. **(D)** PCA analysis divided Wilms tumor samples into two distinct subtypes, Cluster 1 (n=62) and Cluster 2 (n=63). **(E)** The heatmap illustrates the expression patterns of 37 differentially expressed NK cell-related genes across distinct subtypes of Wilms tumor. **(F)** The Kaplan-Meier curves demonstrate the survival differences between the two subtypes (*p* = 0.020). Boxplots display the ESTIMATEScore, ImmuneScore, StromalScore **(G)**, the infiltration levels of 28 immune cell subtypes **(H)**, as well as the expression differences of immune checkpoint genes **(I)** for the two subtypes.

We performed consensus clustering analysis on 125 WT samples using the expression profiles of 37 differentially expressed NRGs and determined the optimal subtype number by the cumulative distribution function (**Figures 3A, B**). The results indicated that the clustering stability was highest at *K*=2, which was further validated by the delta area plot (**Figure 3C**). Subsequently, the 125 WT samples were divided into two subtypes: Cluster 1 (*n*=62) and Cluster 2 (*n*=63). PCA revealed significant differences in gene expression patterns between these subtypes (**Figure 3D**). Heatmap demonstrated distinct relative expression levels of 37 differentially expressed NRGs across the two subgroups (**Figure 3E**). Kaplan-Meier survival analysis showed that patients in Cluster 2 had significantly worse OS compared to Cluster 1 (**Figure 3F**). Immune status assessment using the ESTIMATE and ssGSEA algorithms indicated that WT samples in Cluster 2 exhibited lower ImmuneScore and reduced infiltration levels of multiple immune cell populations (**Figures 3G, H**). Additionally, immune checkpoint-related genes showed marked expression differences between the subtypes (**Figure 3I**). Integrative analysis suggested that WT samples in Cluster 1 tended to exhibit “hot tumor” features, characterized by enhanced immune cell infiltration and more favorable survival outcomes. These findings provide critical insights for exploring personalized therapeutic strategies and prognostic evaluation in WT.

### Screening of TGX-221 and evaluation of its antitumor activity in WT cell lines

**Figure 4:**
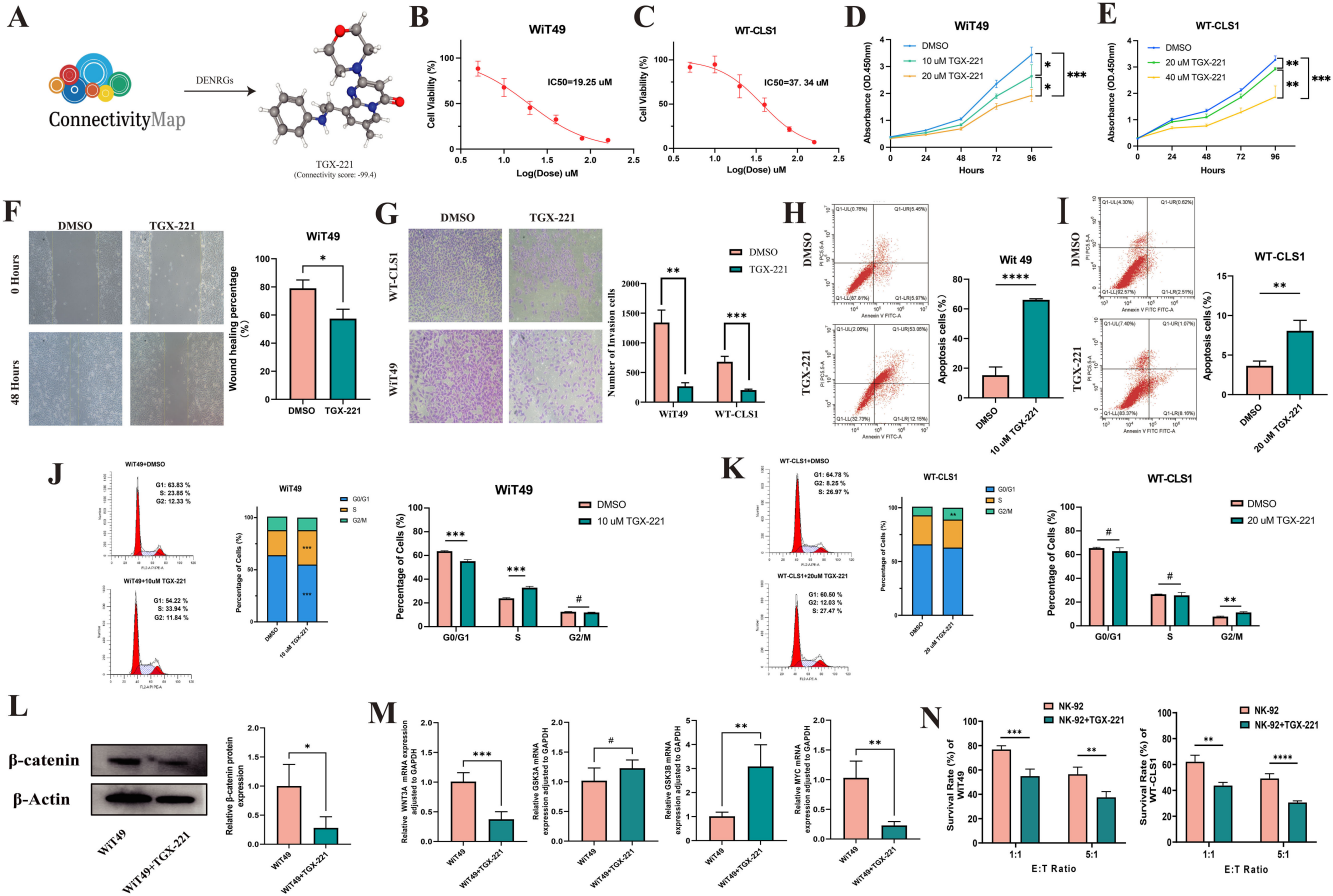
TGX-221 effectively inhibits the malignant phenotype of Wilms tumor. **(A)** Identification of potential small molecule compounds TGX-221 targeting 37 NK cell-related genes using the CMap database. The IC50 values of TGX-221 in WiT49 and WT-CLS1 cell lines are 19.25 µM **(B)** and 37.34 µM **(C)**, respectively. TGX-221 significantly inhibits the proliferation of WiT49 **(D)** and WT-CLS1 **(E)** cells in a dose-dependent manner. The scratch and transwell assays indicate that TGX-221 effectively inhibits the migration of WiT49 cells **(F)** and the invasion of both WiT49 and WT-CL1 cells **(G)**. Annexin V/PI staining demonstrates that TGX-221 induces apoptosis in WiT49 **(H)** and WT-CLS1 cells **(I)**. Cell cycle analysis reveals that TGX-221 induces S-phase arrest in WiT49 cells **(J)** and prolongs the G2 phase in WT-CLS1 cells **(K)**. Changes in β-catenin protein expression levels **(L)** and mRNA expression levels of WNT3A, MYC, GSK3B, and GSK3A after TGX-221 treatment **(M)**. TGX-221 treatment enhances the susceptibility of WiT49 and WT-CLS1 cells to NK cell-mediated cytotoxicity, showing reduced viability compared to untreated cells at effector-to-target ratios of 1:1 and 5:1 **(N)**.

Given the critical role of these 37 differentially expressed NRGs in survival outcomes and immune characteristics across WT subtypes, we screened and identified a small-molecule drug, TGX-221 (**Figure 4A**, **Supplementary File 2**), through the CMap database. This compound is hypothesized to regulate the expression of these genes, potentially promoting an immune-mediated shift from “cold” to “hot” tumor phenotypes, thereby enhancing patient outcomes. *In vitro* experiments revealed concentration-dependent inhibitory effects of TGX-221 on WT cell viability, with IC50 determined for two WT cell lines (**Figures 4B, C**). CCK-8 assays demonstrated that TGX-221 significantly suppressed WT cell proliferation in a dose-dependent manner (**Figures 4D, E**). Scratch wound healing and Transwell assays further confirmed its ability to impair WT cell migration and invasion (**Figures 4F, G**). Flow cytometry analyses showed that TGX-221 effectively induced apoptosis in WT cells (**Figures 4H, I**), while cell cycle profiling revealed S-phase arrest and G2-phase extension as a potential mechanism underlying its antiproliferative effects (**Figures 4J, K**). We observed that the expression of β-catenin protein was significantly downregulated in WiT49 cells treated with TGX-221(**Figure 4L**). Additionally, the mRNA expression of MYC, a downstream target gene of the WNT/β-catenin pathway, was also inhibited. Furthermore, the expression of WNT3A, a classical ligand of the WNT pathway, was suppressed following TGX-221 treatment. TGX-221 promotes the degradation of β-catenin by upregulating the expression of GSK3B (**Figure 4M**). Our results demonstrate that WiT49 and WT-CLS1 incubated with TGX-221 exhibited significantly enhanced susceptibility to NK cell-mediated cytotoxicity, resulting in reduced viability compared to untreated tumor cells, when co-cultured with NK cells across effector-to-target ratios of 1:1 and 5:1 (**Figure 4N**). These findings collectively highlight the robust antitumor activity of TGX-221 in WT cellular models.

### Molecular docking and dynamics simulations of TGX-221 with hub NRGs

**Figure 5:**
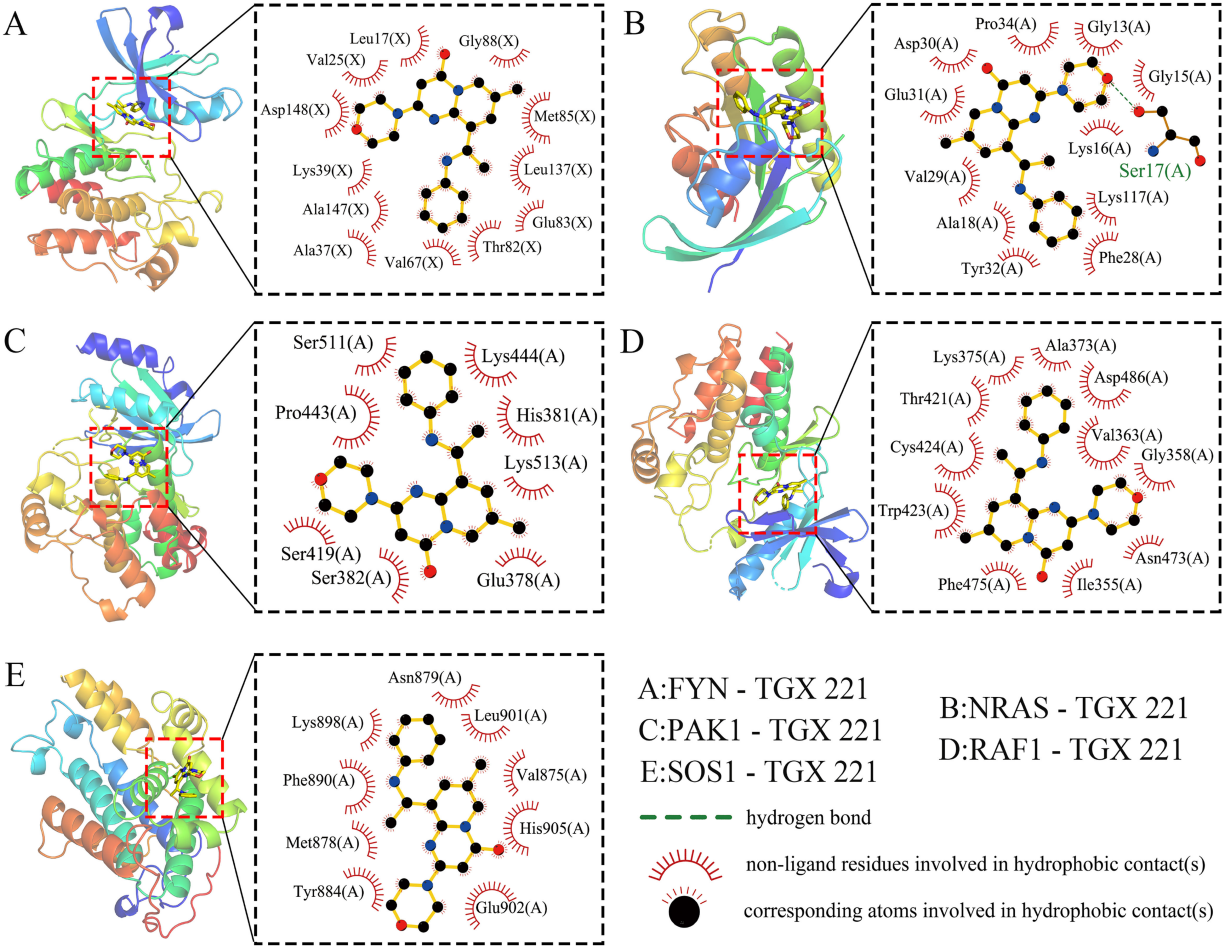
Molecular docking. Molecular docking reveals strong binding affinities between TGX-221 and the target proteins FYN -9.2 kcal·mol^-1^ **(A)**, NRAS -8.4 kcal·mol^-1^ **(B)**, PAK1 -7.1 kcal·mol^-1^ **(C)**, RAF1 -8.6 kcal·mol^-1^ **(D)**, and SOS1 -7.1kcal·mol^-1^ **(E)**.

Given the significant anti-WT activity of TGX-221 observed *in vitro* experiments, we investigated its mechanism of action, focusing on whether its antitumor efficacy is mediated through the regulation of NRGs expression. Using protein-protein interaction network analysis and CytoHubba, we identified 5 hub genes (FYN, NRAS, PAK1, RAF1, and SOS1) from 37 differentially expressed NRGs (**Supplementary Figure 1**). Molecular docking revealed strong binding affinities between TGX-221 and these proteins, with binding energies of -9.2, -8.4, -7.1, -8.6, and -7.1 kcal·mol^-1^ for FYN, NRAS, PAK1, RAF1, and SOS1, respectively (**Figures 5A–E**). Notably, FYN, NRAS, and RAF1 exhibited particularly strong interactions (binding energies below -7.2 kcal·mol^-1^). Binding mode analysis showed that TGX-221 primarily formed hydrophobic interactions with key amino acid residues across all five proteins, while only Ser17 in NRAS formed a hydrogen bond with TGX-221 (**Figure 5C**). This limited hydrogen bonding aligns with TGX-221’s low hydrophilic group content, but the prevalence of hydrophobic interactions ensured robust binding stability.

**Figure 6:**
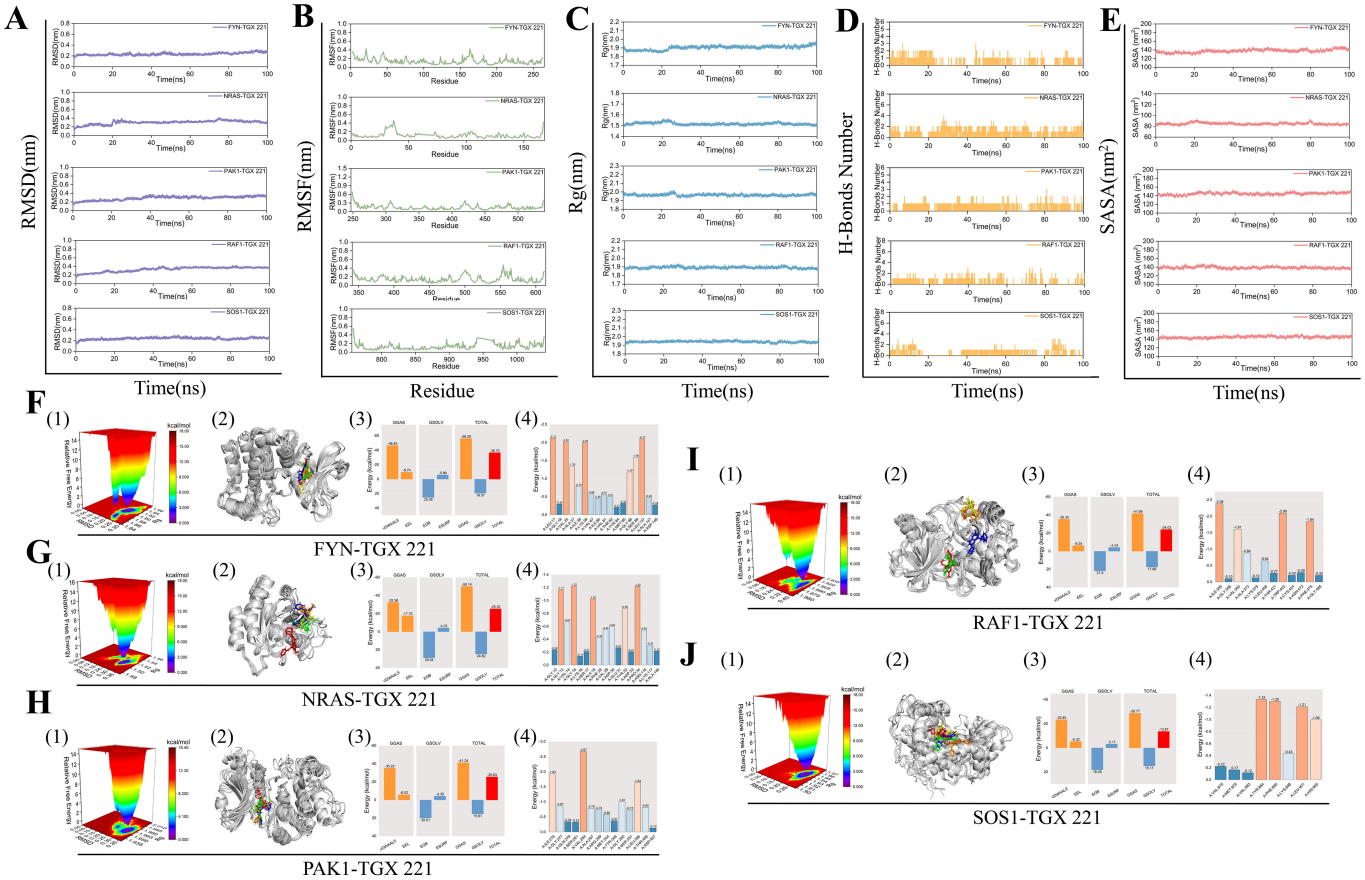
Molecular dynamics simulations. The stability of the FYN, NRAS, PAK1, RAF1, and SOS1 protein complexes with TGX-221 is confirmed by RMSD **(A)**, RMSF **(B)**, Rg **(C)**, hydrogen bond number **(D)**, and SASA **(E)** curve analysis. **(F1, G1, H1, I1, J1)** show the free energy distribution of the complexes formed between FYN, NRAS, PAK1, RAF1, SOS1 proteins, and TGX-221, respectively. **(F2, G2, H2, I2, J2)** present the structural comparisons of these complexes at five-time points (0, 25, 50, 75, and 100 ns) in molecular dynamics simulations, with the red, green, blue, yellow, and orange small molecules corresponding to the structures of TGX-221 at different time points. **(F3, G3, H3, I3, J3)** list the average binding free energies of the respective complexes, where VDWAALS, EEL, EGB, ESURF, GGAS, GSOLV, and TOTAL represent the van der Waals interactions, electrostatic energy, polar solvation energy, non-polar solvation energy, molecular mechanics term, solvation energy term, and the total average binding free energy, respectively. **(F4, G4, H4, I4, J4)** reveal the energy contributions of the amino acid residues in each protein that participate in the binding with TGX-221.

To validate the binding affinity and stability of TGX-221 with its target proteins, we conducted 100 ns molecular dynamics simulations on the complexes of TGX-221 with FYN, NRAS, PAK1, RAF1, and SOS1. As shown in **Figures 6A-E**, stability analysis revealed that RMSD curves remained stable within 1 nm, RMSF curves showed minimal amino acid movement (<1 nm), and Rg curves indicated compact protein structures (1.5–2.0 nm). SASA curves fluctuated steadily with values of 140 nm² (FYN), 80 nm² (NRAS), 140 nm² (PAK1), 140 nm² (RAF1), and 140 nm² (SOS1); the narrow fluctuation range for NRAS was attributed to its smaller size. Hydrogen bonds were stable (1–2), with predominant hydrophobic interactions. Conformational analysis showed that TGX-221 maintained stable binding with most proteins, although RAF1’s binding site underwent minor changes without detachment (**Figures 6F-H**). Free energy landscape analysis indicated that the SOS1-TGX-221 complex formed a single minimum energy cluster, suggesting the most stable binding. In contrast, the complexes of FYN, NRAS, PAK1, and RAF1 formed two clusters, transitioning from an initial stable state to a more stable state (**Figures 6F-H**). Binding free energy calculations revealed that FYN exhibited the strongest binding (-36.72 kcal·mol^-1^), while the binding energies of the other proteins ranged from -25.32 to -13.67 kcal·mol^-1^ (**Figures 6F-H**). Key residues contributing to binding affinity included FYN (LEU-17, LEU-137, VAL-125, LYS-39), NRAS (GLY-15, PRO-34), PAK1 (VAL-284, ILE-276, LEU-396), RAF1 (LEU-355, TRP-423, PHE-475), and SOS1 (TYR-884, PHE-890, LEU-901) (**Figures 6F-H**). Overall, these results confirmed that TGX-221 formed stable complexes with these proteins, particularly with FYN, where key residues played critical roles in binding. These interactions suggest that TGX-221 may significantly influence the structural and functional properties of these proteins, thereby contributing to its observed anti-WT effects.

### Construction and validation of a prognostic signatures for WT based on 37 differentially expressed NRGs

**Figure 7:**
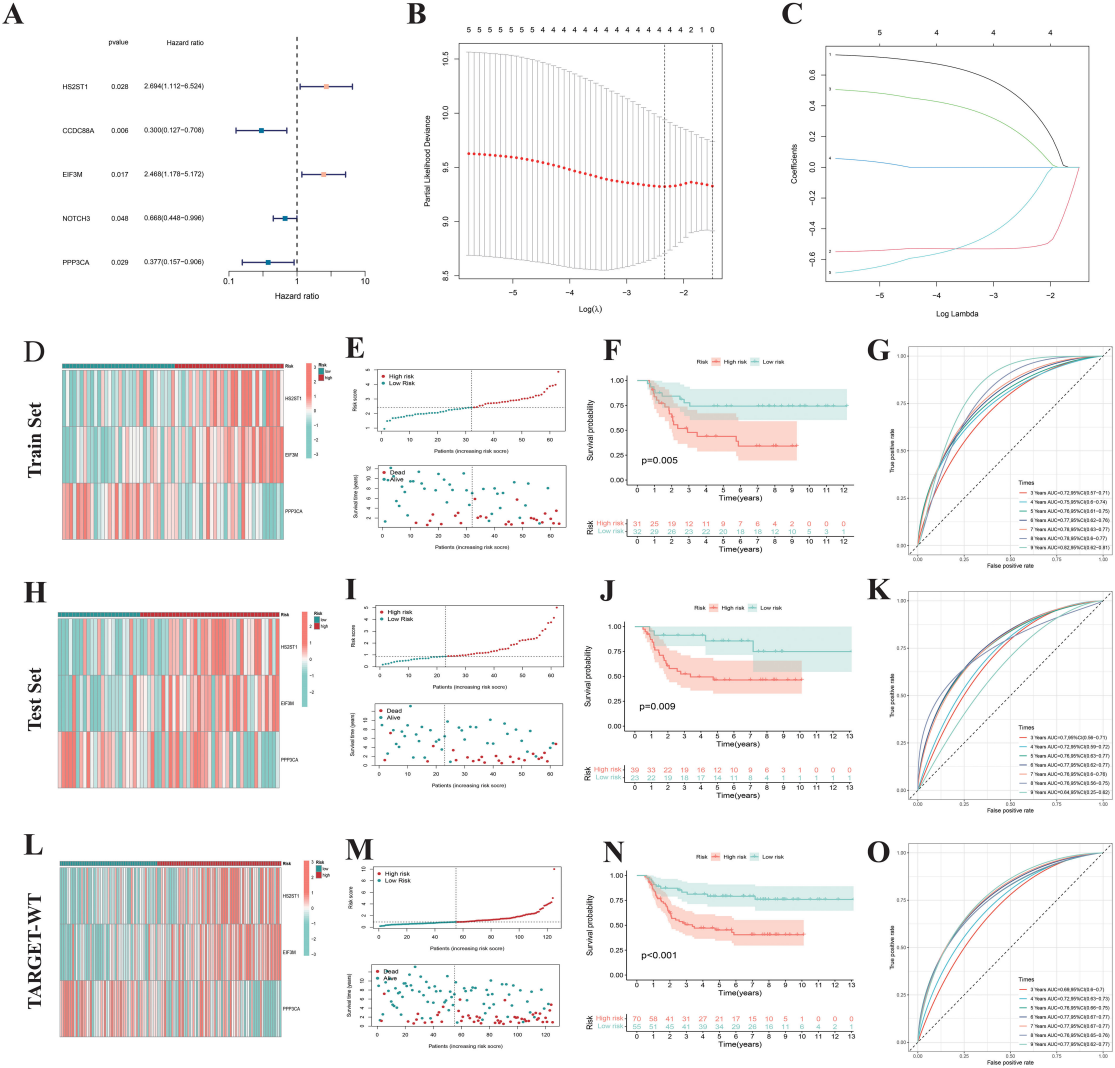
Construction of prognostic signatures. **(A)** Cox regression analysis of 37 NK cell-related genes identified 5 prognostic genes. **(B, C)** Prognostic signature constructed via Lasso regression. **(D)** Heatmap showing expression patterns of 3 risk genes across different risk subgroups. **(E)** Survival status distribution indicates that the number of deaths in Wilms tumor patients increases with higher risk scores. **(F)** The K-M survival curve also shows that Wilms tumor patients with high-risk scores have poorer overall survival. **(G)** The area under the ROC curve validates the predictive performance of the prognostic signatures. Similar results were obtained in the Test set **(H–K)** and TARGET-WT cohort **(L–O)**.

Given that only the TARGET-WT cohort currently has complete prognostic information among WT research cohorts, we conducted prognostic analyses on 125 WT samples from this cohort, randomly dividing them into training set (63 cases) and test set (62 cases). In the training set, univariate Cox regression analysis on 37 differentially expressed NRGs identified five NRGs— HS2ST1, CCDC88A, EIF3M, NOTCH3, and PPP3CA — significantly associated with OS in WT patients (**Figure 7A**). Subsequently, LASSO regression analysis using 10-fold cross-validation identified three genes (HS2ST1, EIF3M, and PPP3CA) with significant prognostic risk (**Figures 7B, C**). A prognostic signatures model was constructed, with risk coefficients determined by multivariate Cox regression (HS2ST1: 0.773, EIF3M: 0.645, PPP3CA: -0.927). Using these coefficients and gene expression levels, we calculated a risk score for each WT sample, dividing them into high-risk and low-risk groups based on the median risk score of the training set. The heatmap revealed higher expression of HS2ST1 and EIF3M in the high-risk subgroup WT samples (**Figure 7D**). The survival status distribution plot indicates that the number of deaths among WT patients significantly increases with higher risk scores (**Figure 7E**). K-M survival analysis confirmed that low-risk patients had significantly better survival outcomes (**Figure 7F**), consistent across WT subtypes with varying clinical characteristics (**Supplementary Figure 2**), demonstrating the robustness and broad applicability of the prognostic signatures model. ROC curve analysis showed AUC values ranging from 0.72 to 0.82 for 3- to 9-year survival predictions, indicating high predictive accuracy (**Figure 7G**). These results were validated in both the test set (**Figures 7H-K**) and the complete TARGET-WT cohort (**Figures 7L-O**), confirming the model’s reliability and effectiveness.

### Construction and validation of nomogram

**Figure 8:**
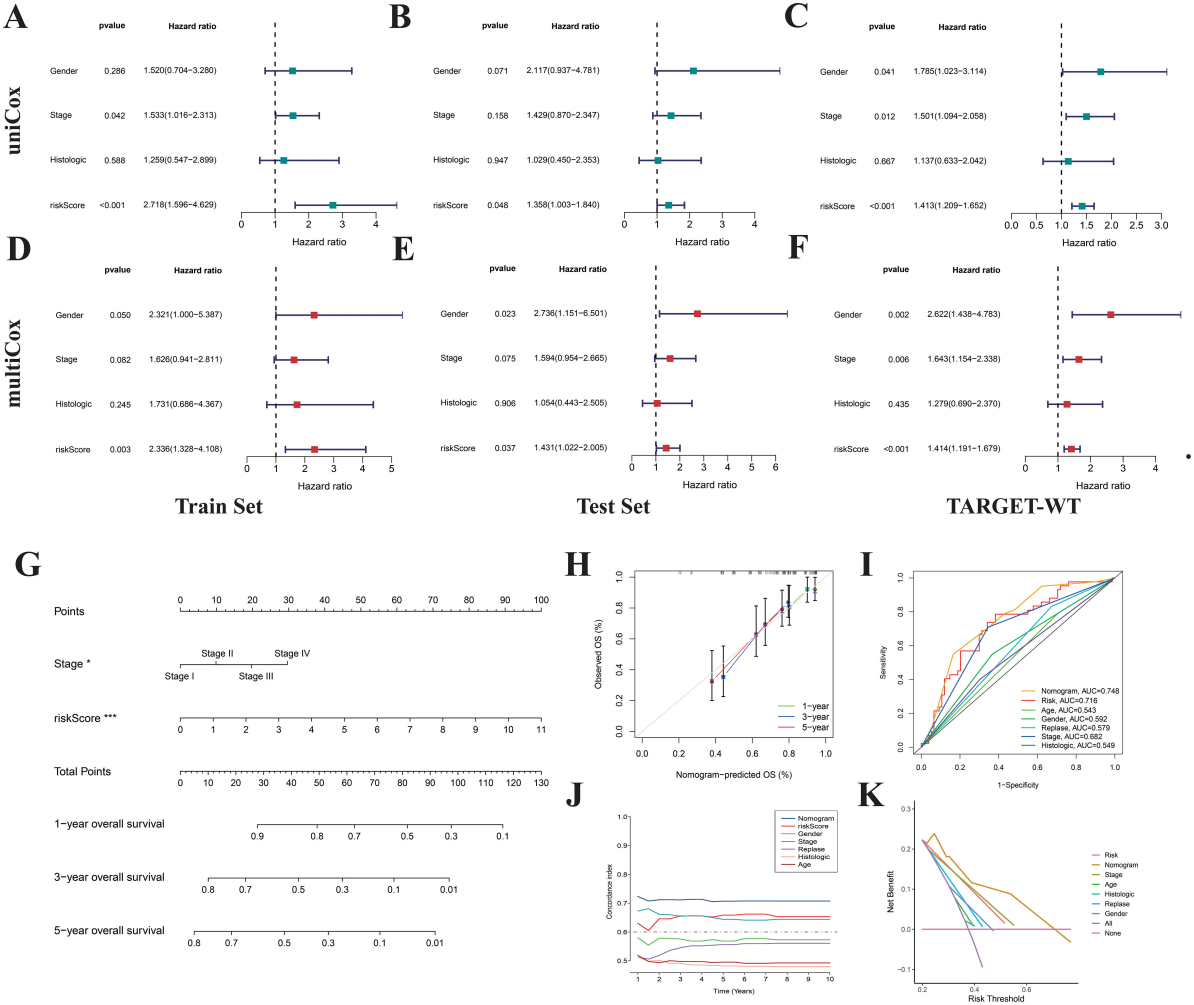
Construction of nomogram. Univariate **(A-C)** and multivariate **(D-F)** Cox regression analyses indicate that the risk score is an independent prognostic factor for Wilms tumor. **(G)** Nomogram constructed based on risk score and stage. Calibration curves **(H)**, ROC curves **(I)**, concordance index **(J)**, and DCA curves **(K)** were used to evaluate the accuracy and sensitivity of the nomogram’s prognostic predictions.

Through both univariate and multivariate Cox regression analyses based on the training set, test set, and the complete TARGET-WT cohort, we identified the risk score as an independent predictor of prognosis in WT patients, unaffected by other clinical characteristics (**Figure 8A-F**). To facilitate the clinical application of the prognostic signatures, we developed a nomogram based on patient staging and risk score (**Figure 8G**). This nomogram enables precise quantification of WT patient OS probabilities at 1, 3, and 5 years. Calibration curves demonstrated that the nomogram’s predicted values were highly consistent with observed survival probabilities (**Figure 8H**), confirming its excellent predictive accuracy. ROC curve analyses revealed that the nomogram (AUC=0.748) outperformed the risk score (AUC=0.716) and clinical features alone in survival prediction (**Figure 8I**), showcasing its higher specificity and predictive accuracy. Additionally, consistency index and decision curve analyses further demonstrated the nomogram’s maximal net benefit in clinical decision-making (**Figures 8J, K**). These findings indicate that the NRGs-based prognostic signatures and nomogram offer superior clinical utility compared to traditional clinical models, providing more accurate and quantitative prognostic assessments.

### Characterization of immune and mutation landscape in WT risk subgroups

**Figure 9:**
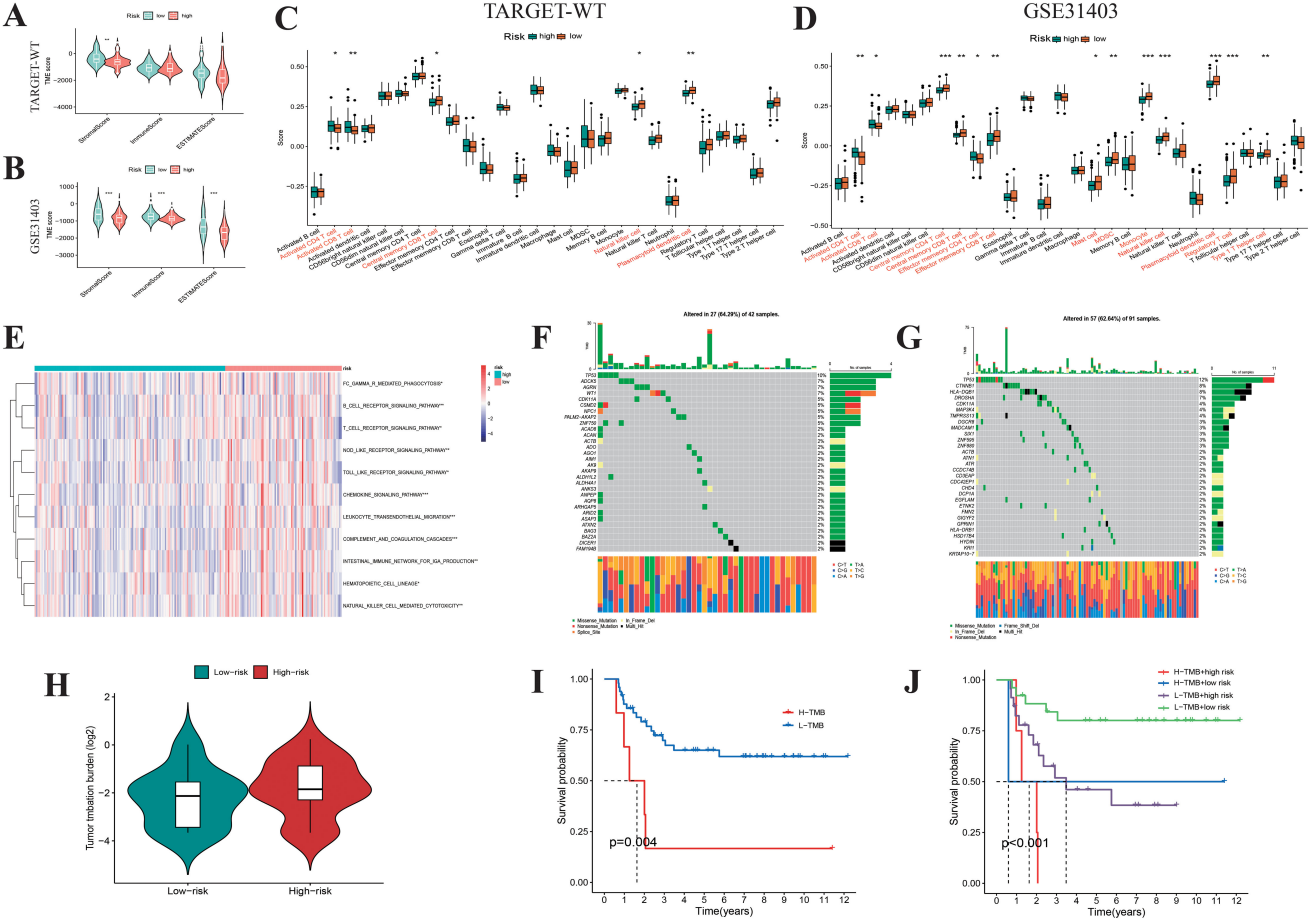
Biological feature analysis of Wilms tumor samples with high- and low-risk scores. Boxplots display the ESTIMATEScore, ImmuneScore, StromalScore **(A, B)**, and the infiltration levels of 28 immune cell subtypes **(C, D)** between high- and low-risk Wilms tumor samples. **(E)** GSVA analysis shows the enrichment scores of multiple immune pathways in high- and low-risk Wilms tumor samples. **(F, G)** Waterfall plot showing the mutational spectrum of genes in high- and low-risk Wilms tumor samples. **(H)** Violin plots compare the differences in tumor mutation burden between high- and low-risk Wilms tumor samples. **(I, J)** K-M survival curves show the relationship between tumor mutation burden and risk score with overall survival in Wilms tumor samples.

Using the ESTIMATE analysis, we found that low-risk WT patients had significantly higher immune scores compared to high-risk patients, as validated by the external dataset GSE31403 (**Figures 9A, B**). Quantification of 28 immune cell subtypes via the ssGSEA algorithm revealed significant differences between high-risk and low-risk subgroups, with low-risk WT samples showing higher infiltration of NK cells and plasmacytoid dendritic cells (**Figures 9C, D**). Pathway enrichment analysis identified activation of immune-related pathways in low-risk WT samples (**Figure 9E**), including NK cell-mediated cytotoxicity, T-cell receptor signaling, and chemokine signaling, suggesting low-risk WT samples have higher immune activity and better survival outcomes. For genetic mutations, TP53 and ADCK5 mutations were more frequent in the low-risk group, while TP53 and CTNNB1 mutations were more prevalent in the high-risk group (**Figures 9F, G**). The high-risk group also exhibited higher tumor mutation burden (TMB) (**Figure 9H**), with high TMB linked to poorer survival outcomes (**Figures 9I, J**). These findings suggest that mutations driving abnormal tumor cell proliferation and immune evasion, such as CTNNB1 mutations leading to unchecked cell cycle progression and Wnt pathway activation, contribute to these outcomes. Overall, classifying WT samples based on risk scores provides insights into distinct immune and mutational profiles, supporting precision medicine and personalized treatment strategies.

### Investigation of HS2ST1, EIF3M, and PPP3CA in immune modulation within WT and their potential as targets for immune therapy

**Figure 10:**
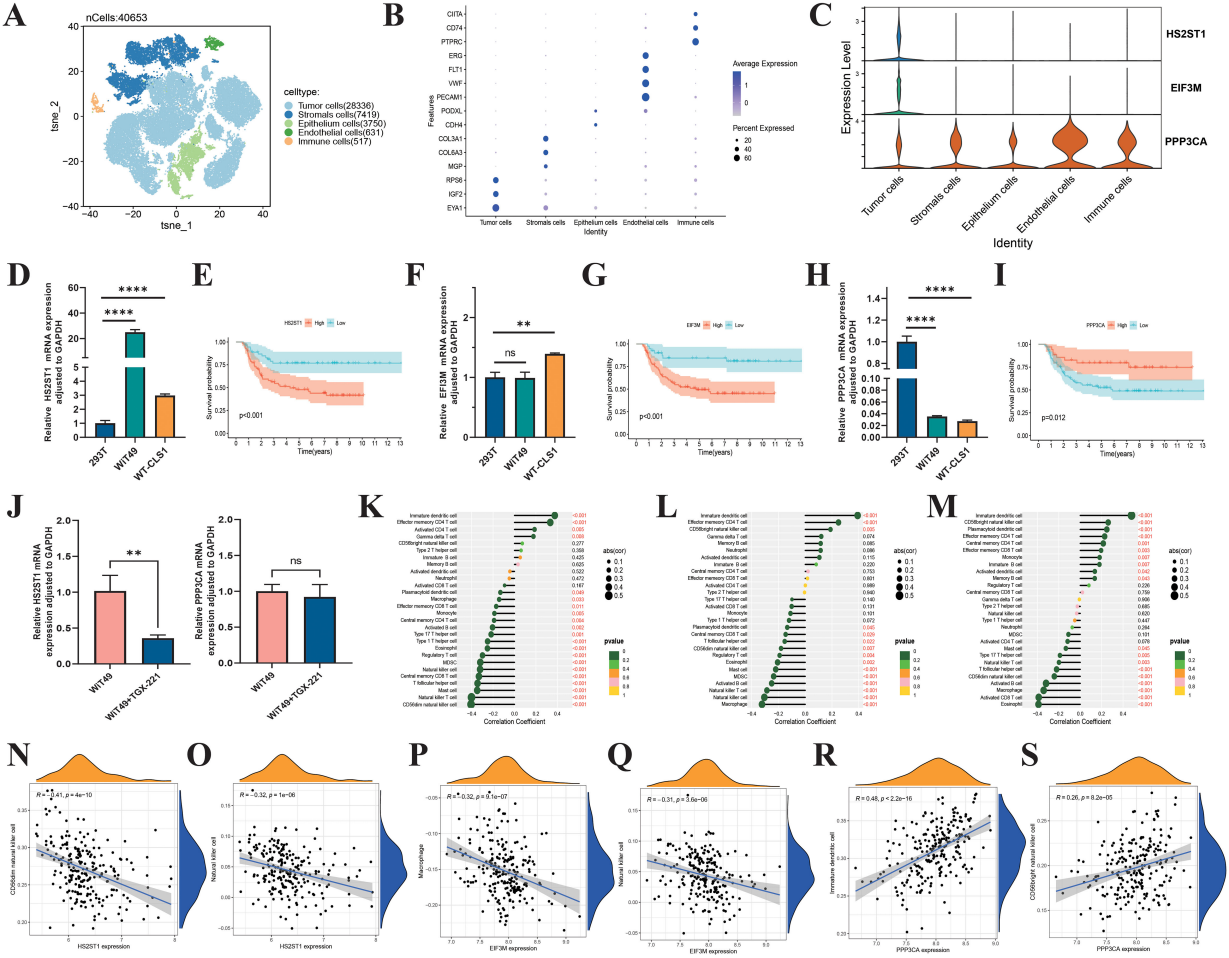
Biological roles of HS2ST1, EIF3M, and PPP3CA in Wilms tumor. **(A)** UMAP analysis of single-cell RNA sequencing data from six Wilms tumor samples clearly shows the distribution of 40,654 cells. **(B)** These cells are categorized into 5 major clusters: Tumor, Stromal, Epithelium, Endothelial, and Immune, each identified by specific marker genes. **(C)** Violin plots show the expression levels of 3 risk genes across different cell types in the Wilms tumor. RT-qPCR validation of the expression levels of HS2ST1 **(D)**, EIF3M **(F)**, and PPP3CA **(H)**. K-M survival curves evaluate the impact of HS2ST1 **(E)**, EIF3M **(G)**, and PPP3CA **(I)** expression levels on overall survival in Wilms tumor. **(J)** HS2ST1mRNA expression levels in tumor cells following TGX-221 treatment. **(K–S)** Correlation analysis between the expression levels of risk genes and the infiltration of 22 immune cell subtypes in Wilms tumor.

After data quality control, filtering, batch correction, dimensionality reduction, and clustering, we identified 40,653 cells and annotated them into 5 major subtypes: tumor cells, stromal cells, epithelial cells, endothelial cells, and immune cells (**Figures 10A, B**). HS2ST1 and EIF3M were predominantly expressed in tumor cells, while PPP3CA was mainly expressed in non-tumor cells, such as immune cells (**Figure 10C**). We validated these findings using RT-qPCR, which showed that HS2ST1 and EIF3M were significantly upregulated in WT cell lines (WiT49 and WT-CLS1), whereas PPP3CA exhibited significant expression in human normal renal epithelial cells (293T) (**Figures 10D, F, H**). K-M survival analysis demonstrated a close correlation between the expression of these genes and patient outcomes (**Figures 10E, G, I**). Interestingly, we also found that TGX-221 effectively inhibits the expression of HS2ST1 (**Figures 10J**). Pearson correlation analysis revealed significant associations between the expression levels of these genes and the infiltration of various immune cells (**Figures 10K-M**). Specifically, HS2ST1 and EIF3M expression levels were significantly negatively correlated with the infiltration of CD56dim NK cells, while PPP3CA expression was significantly positively correlated with the infiltration of CD56bright NK cells and immature dendritic cells (**Figures 10N-S**). Collectively, HS2ST1, EIF3M, and PPP3CA may play crucial roles in the development and progression of WT. HS2ST1 and EIF3M likely influence the tumor immune microenvironment by inhibiting NK cell function, whereas PPP3CA may exert protective effects by promoting the infiltration of specific immune cells. These distinct expression patterns and regulatory roles in the immune microenvironment provide a strong basis for considering these genes as potential immunotherapy targets in WT.

## Discussion

WT, the most prevalent embryonal malignancy in the pediatric urinary system, has witnessed improved survival rates through current diagnostic frameworks that incorporate histopathological features (e.g., anaplastic vs. non-anaplastic subtypes) and monogenic molecular subtyping (3, 8, 14). Nevertheless, high-risk subtypes characterized by tumor heterogeneity-driven relapse and chemoresistance persist as clinical challenges, a predicament rooted in conventional classification systems’ inadequacy to resolve the dynamic evolution of the tumor microenvironment (42). Emerging evidence implicates that NK cells, innate immune effector cells, participate in the immune editing process of solid tumors through their cytotoxic effects and immune regulatory functions (23, 28). However, the specific mechanisms of NK cells in WT remain unclear. This study elucidates the infiltration characteristics of NK cells in the tumor microenvironment of WT, provides a new perspective for understanding the immune evasion mechanisms of WT, and may offer a theoretical basis for NK cell-based immunotherapy for WT.

Based on the analysis of the CQMU cohort and the TARGET-WT database, this study found a significant deficiency in immune infiltration in WT tissues compared to normal kidney tissues, with a particularly notable reduction in the infiltration level of the CD56dim NK cell subset. This finding suggests that NK cell dysfunction may be a key microenvironmental feature driving the malignant progression of WT. Through integrated multi-omics analysis, this study systematically characterized the gene expression patterns related to NK cells in WT. The cross-cohort analysis identified 37 differentially expressed NRGs with significant copy number variations, among which copy number gains in genes such as CREB3L4 may influence NK cell function remodeling by interfering with MAPK signaling transduction (43, 44). Consensus clustering based on differentially expressed NRGs for the first time stratified WT into two molecular subtypes with significant prognostic differences. Cluster 1 exhibited a “hot” tumor phenotype, characterized by elevated immune scores and increased infiltration of CD8+ T cells and NK cells, which corresponded to better survival outcomes. In contrast, Cluster 2 presented an “immune desert” phenotype, with suppressed expression of immune checkpoint molecules and poor clinical outcomes. This novel classification system, based on the dynamic evolution of the tumor microenvironment, overcomes the limitations of traditional histopathological classification in identifying high-risk subtypes and provides a molecular basis for the precise selection of clinical treatment strategies. This new classification system better reflects the biological behavior and immune status of the tumor, and has the potential to improve prognostic assessment and treatment response prediction in WT patients.

The advent of combinatorial immunotherapeutics opens new avenues for WT management (45, 46). Preclinical evidence supports the synergistic efficacy of immune checkpoint inhibitors paired with CAR-T or NK cell-based therapies (25–27, 47). Targeting Cluster 2’s immune-cold phenotype using such strategies may counteract immune microenvironmental resistance mechanisms. Future trials should prioritize validating these combinatorial regimens in high-risk WT subtypes to amplify therapeutic efficacy while mitigating off-target toxicity.

In the realm of translational medicine, this study identified through computational pharmacology that the PI3Kβ-specific inhibitor TGX-221 may potentially reverse the expression profiles of NRGs in high-risk subtypes (48–51). Molecular docking and dynamics simulations revealed that TGX-221 forms stable complexes with hub NRG targets such as FYN (binding energy -36.72 kcal·mol^-1^) (52, 53). Its mechanism of action may involve the inhibition of the PI3K/AKT pathway and the activation of the MAPK/ERK signaling axis, thereby remodeling the TME in WT (54, 55). *In vitro* experiments confirmed that TGX-221 may significantly attenuate the malignant phenotype of WT cells by inducing S-phase arrest and inhibiting the epithelial-mesenchymal transition process. These findings suggest that the compound may achieve an immune phenotypic shift from “cold” to “hot” tumors in high-risk WT through a dual mechanism of direct cytotoxic effects and remodeling of the tumor immune microenvironment offering a new strategy to overcome chemoresistance in conventional chemotherapy. However, despite its promising antitumor potential demonstrated *in vitro*, the efficacy and safety of TGX-221 in clinical applications still require further validation. Future research should focus on the *in vivo* pharmacological evaluation of TGX-221 and its potential for combination with other immunotherapeutic agents. For example, combining TGX-221 with immune checkpoint inhibitors or NK cell therapy may further enhance its antitumor efficacy. Additionally, considering the successful application of combination therapies in various types of tumors, such as non-small cell lung cancer and breast cancer, similar strategies could be explored for WT in the future, to provide more effective treatment options for patients with WT.

The essence of constructing a predictive model is to transform the elucidation of molecular mechanisms into clinically actionable decision-making tools through systems biology approaches. This not only identifies biomarkers associated with prognosis but also more accurately predicts patients’ survival, recurrence risk, and treatment response. For example, a prognostic model based on immune-related lncRNAs can effectively predict the prognosis of colorectal cancer patients and the efficacy of chemotherapeutic drugs, providing new biomarkers and therapeutic targets for precision treatment in colorectal cancer (56). In this study, a prognostic model was constructed based on differentially expressed NRGs. Through LASSO-Cox regression analysis, key variables such as HS2ST1, EIF3M, and PPP3CA were identified. A risk score was calculated for each WT patient, and patients were stratified into high- and low-risk score groups. Patients with high-risk scores exhibited poorer clinical outcomes. This NRGs-related prognostic model overcomes the limitations of traditional clinical parameters (such as tumor stage and histological classification) in predicting outcomes. This quantitative stratification can guide clinical practice: for example, in low-risk scores WT patients, overtreatment (such as reducing chemotherapy cycles) can be avoided to minimize long-term complications (such as secondary tumors and cardiopulmonary toxicity). In contrast, high-risk scores WT patients should undergo intensified follow-up (e.g., reducing the interval of imaging surveillance from every 6 months to every 3 months) and be prioritized for inclusion in targeted therapy clinical trials (such as TGX-221).

The risk genes within the prognosis model have the potential to serve as novel biomarkers for WT. For instance, single-cell analysis revealed that the oncogenic genes HS2ST1 and EIF3M are primarily expressed in tumor cells, while the protective gene PPP3CA is mainly expressed in immune cells. This was also confirmed by PCR results. These risk genes are not isolated predictive factors but rather key genes in constructing a predictive network, each with its biological functions. For example, HS2ST1 may enhance Wnt/β-catenin signaling by mediating heparan sulfate modification, thereby promoting tumor stem cell properties; EIF3M may regulate the translation efficiency of oncogenic proteins through the eIF3 complex; and PPP3CA, as the catalytic subunit of calcineurin, may inhibit NK cell IL-2/IFN-γ secretion capacity when its expression is downregulated due to impaired NFAT dephosphorylation (57–63). In brief, the prognostic model constructed in this study integrates a molecular network comprising HS2ST1 (oncogenic), EIF3M (translational regulation), and PPP3CA (immune activation). It transforms the heterogeneity of WT into a quantifiable precision stratification and prognostic assessment tool. This prognosis model reveals the potential deep mechanisms of the interaction in the immune microenvironment, explains the differences in clinical outcomes, and provides a bridge for the transition from conventional chemotherapy to individualized, molecularly stratified therapies.

In summary, this study elucidated the dynamic expression patterns of NKGs in WT’s tumor microenvironment and proposed a novel molecular classification system based on immune microenvironment heterogeneity: “immune rich” (Cluster 1) and “immune desert” (Cluster 2). This classification overcomes the limitations of traditional histopathological methods. For the first time, through computational pharmacology screening combined with molecular dynamics simulations, we identified that the PI3Kβ inhibitor TGX-221 could target and regulate the proteins of FYN and NRAS, elucidating its potential mechanism in reversing the immune-suppressive microenvironment. Additionally, based on the LASSO-Cox regression model, we constructed an NRGs risk score model to achieve quantitative stratification of WT prognosis, providing a new tool for clinical individualized treatment (e.g., de-escalation of chemotherapy for low-risk scores patients and targeted intervention for high-risk scores patients). However, this study still has the following limitations: the sample size and diversity of the self-built cohort and public datasets are insufficient, and there is a lack of independent validation across different ethnicities and age groups, which may affect the generalizability of the classification system. The key mechanisms, such as the immune microenvironment remodeling by TGX-221, lack *in vivo* experiments and direct validation of NK cell function, resulting in an incomplete evidence chain for therapeutic efficacy. The stability of the prognostic model has not been tested in external cohorts, and the WT-specific transformation pathways for combining TGX-221 with immunotherapy remain unclear. Future work should focus on expanding cohorts, deepening mechanistic validation, and conducting prospective clinical trials to promote the translation of these findings into clinical practice.

## Conclusion

This study innovatively classified WT patients based on differential expression NKGs, proposing two subtypes: “hot tumor” (immune-rich) and “cold tumor” (immune-desert), thereby overcoming the limitations of traditional histopathological classification. Molecular docking and dynamics simulations revealed that the small-molecule compound TGX-221 can target and modulate key proteins such as FYN and NRAS, potentially reversing the immune-desert microenvironment in WT. Additionally, the constructed NRGs risk score model enables quantitative prediction of WT prognosis, offering a new tool for personalized treatment strategies. Notably, the risk gene HS2ST1 shows potential as a novel biomarker for WT, possibly promoting tumor stem cell properties through the regulation of the Wnt/β-catenin signaling pathway. Future research will further explore the clinical translation potential of these findings to advance the precision treatment of WT.

## Data Availability

The original contributions presented in the study are included in the article/**Supplementary Material**. Further inquiries can be directed to the corresponding authors.
