# Supplementary figures and images for: NK cell-related genes-driven novel molecular subtyping and prognostic signatures for Wilms tumor: uncovering the therapeutic potential of TGX-221 and biomarker role of HS2ST1
Source: Front Oncol. 2025 Aug 28;15:1593011. doi: 10.3389/fonc.2025.1593011 (PMC12422919; doi:10.3389/fonc.2025.1593011)

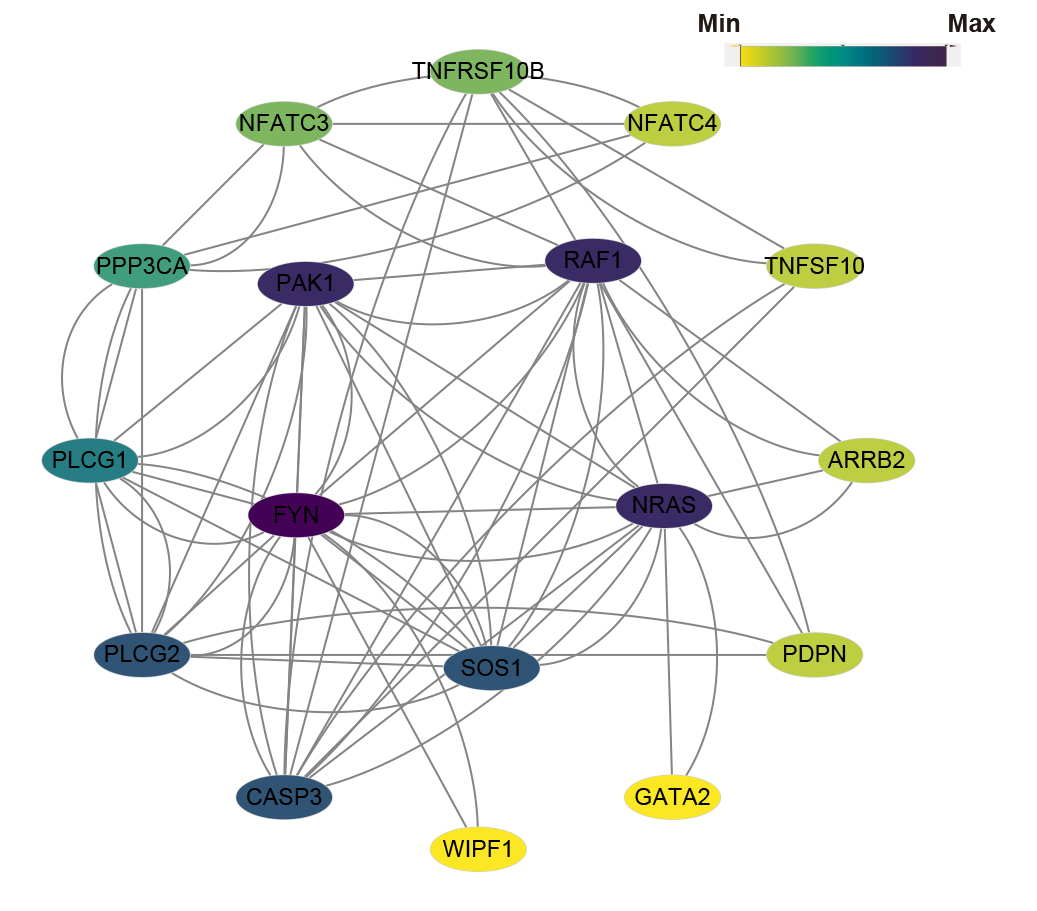

Supplement: Supplementary file 1 [file Image1.jpeg]

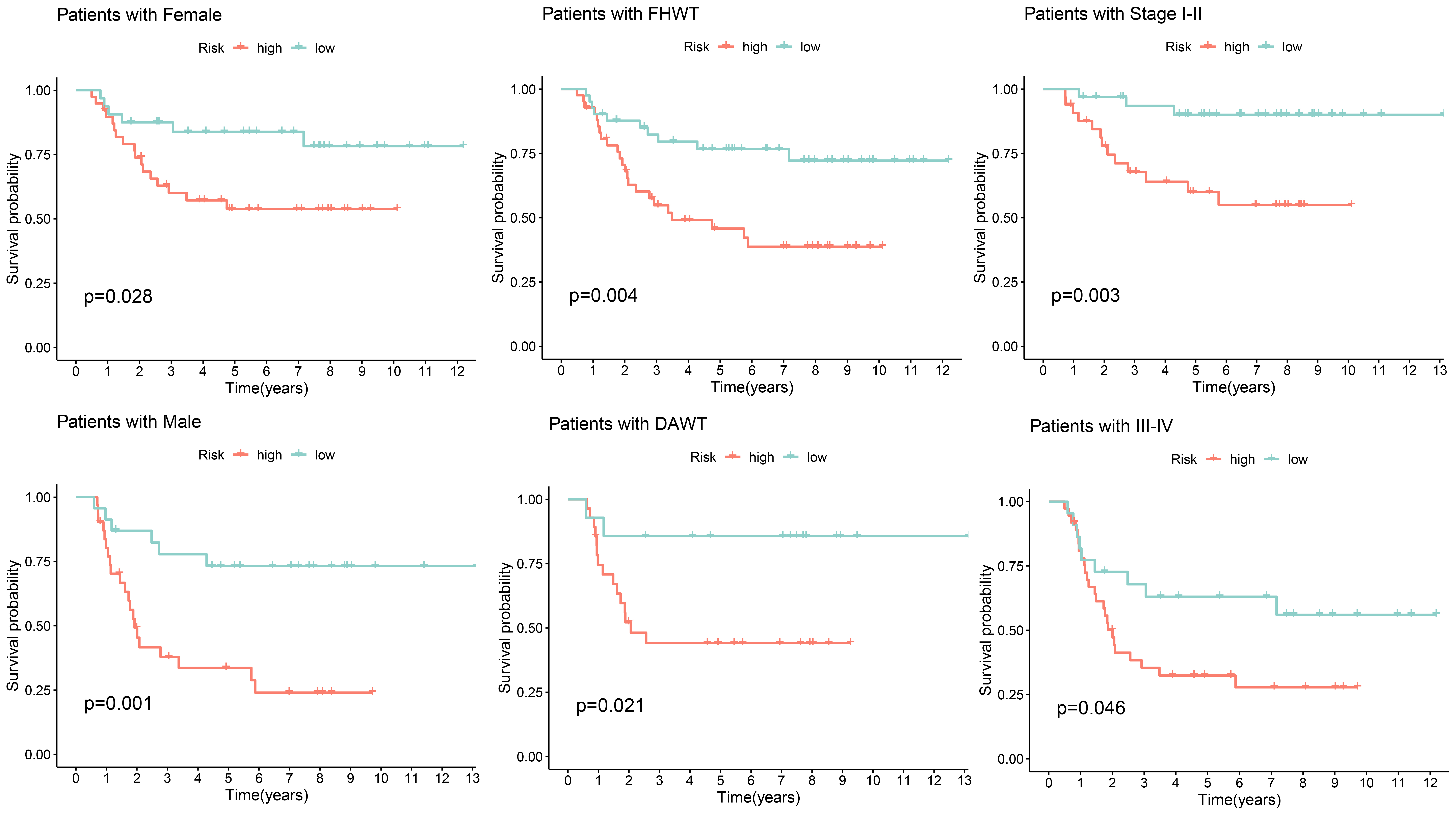

Supplement: Supplementary file 2 [file Image2.jpeg]
